# Syndrome de régression caudale: à propos d’un cas

**DOI:** 10.11604/pamj.2018.30.219.8691

**Published:** 2018-07-19

**Authors:** Charaf Tilfine, Fatima Zahrae Laamrani, Rachida Dafiri

**Affiliations:** 1 Service de Radiologie, Hôpital d’Enfants-Maternité Avicenne, Rabat, Maroc

**Keywords:** Syndrome de régression caudale, agénésie sacrée et coccygienne, syndrome malformatif, Caudal regression syndrome, agenesis of sacral and coccygeal vertebrae, malformative syndrome

## Abstract

Le syndrome de régression caudale est un syndrome malformatif rare associant à des degrés variables une agénésie des vertèbres sacrées et coccygiennes, avec raccourcissement des membres inférieurs, et anomalies gastro-intestinales, génito-urinaires et cardiovasculaires. Sa relation avec le diabète maternel est bien établie. Mais sa cause exacte reste toujours mal établie. Nous présentons un cas rare de ce syndrome chez un nourrisson de 8 mois dont la mère est connue diabétique, et qui présente en plus du syndrome polymalformatif, une constipation chronique.

## Abstract (en)

Caudal regression syndrome is a rare malformative syndrome associated, to varying degrees, with agenesis of sacral and coccygeal vertebrae, lower limb shortening and gastrointestinal, genitourinary and cardiovascular abnormalities. Its relationship with gestational diabetes is well established, but its exact cause is poorly established. We here report a rare case of caudal regression syndrome in a 8-month old infant of diabetic mother with polymalformative syndrome and chronic constipation.

## Introduction

Le syndrome de régression caudale est un syndrome malformatif rare associant à des degrés variables une agénésie des vertèbres sacrées et coccygiennes, avec raccourcissement des fémurs, et anomalies gastro-intestinales, génito-urinaires et cardiovasculaires. Son incidence est de 1 à 5 cas pour 100.000 naissances. Sa cause précise n’est pas encore identifiée mais sa relation avec le diabète maternel est bien établie [1]. Nous présentons un cas rare de ce syndrome chez un nourrisson de 8mois dont la mère est connue diabétique.

## Patient et observation

**Figure 1:**
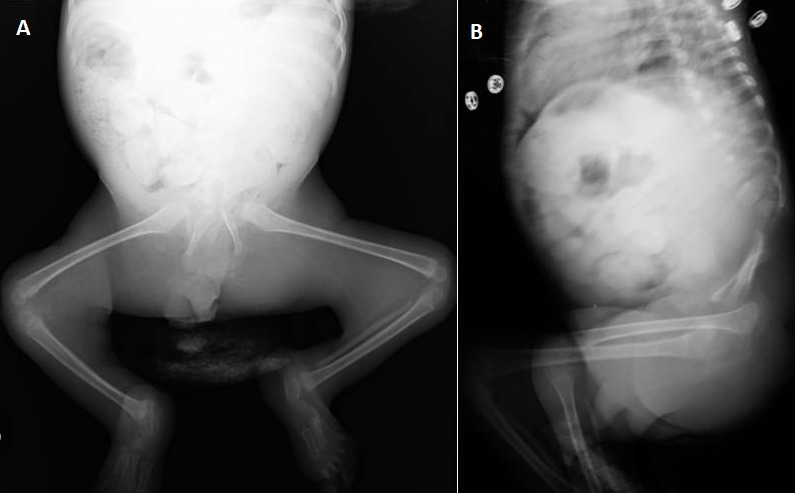
(A) radiographie standard de face du bassin et des membres inférieurs montrant un bassin rétréci en entonnoir avec raccourcissement des fémurs, abduction des cuisses et flexion des genoux réalisant la position en « frog-like » des membres inférieurs; (B) radiographie du rachis dorso-lombo-sacré de profil met en évidence une agénésie sacro-coccygienne et de la dernière vertèbre lombaire

**Figure 2:**
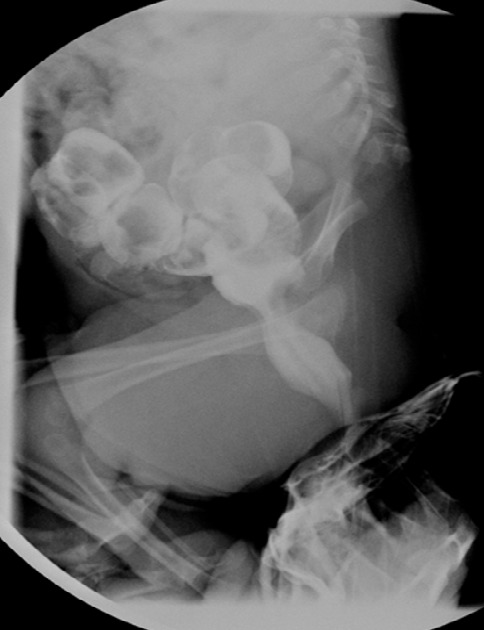
Index baryté: défaut d’expansion rectal siégeant à 5cm de la marge anale, étendu sur 2cm, bien centré, sans distension d’amont mais avec un retard d’évacuation

Il s’agit d’un nourrisson de 8 mois, de sexe masculin, né d’une grossesse mal suivie admis dans notre formation pour syndrome poly malformatif et constipation chronique. Dans les antécédents, on ne retrouve pas de notion de consanguinité, ni de malformations dans la fratrie. En revanche on note un diabète de type II chez la mère qui est mal équilibré. L’examen clinique trouve un nourrisson en bon état général, présentant des malformations de la moitié inférieure du corps à type d’hypotrophie des cuisses avec abduction des hanches et flexion irréductible des genoux, réalisant un aspect « frog-like » de l’extrémité inférieure du corps. Il s’y associe un pertuis cutané dorsal d’un sinus dermique. Les organes génitaux externes, l’anus, les membres supérieurs ainsi que la face sont de morphologie normale. Une radiographie standard de face du bassin et des membres inférieurs réalisée (Figure 1A) a objectivé un bassin rétréci en entonnoir avec raccourcissement des fémurs, abduction des cuisses et flexion des genoux réalisant la position en « frog-like » des membres inférieurs. La radiographie du rachis dorso-lombo-sacré de profil (Figure 1B) a mis en évidence une agénésie sacro-coccygienne et de la dernière vertèbre lombaire sans autres anomalies de segmentation du rachis sus jacent à l’agénésie. Vu les résultats de l’examen clinique ainsi que la radiographie standard, le diagnostic de syndrome de régression caudale a été retenu. Un index baryté (Figure 2) a été réalisé en raison d'une part de la constipation chronique présentée par le nourrisson et d'autres part de la fréquence des malformations ano-rectales associées à l’agénésie sacrée, a révélé un défaut d’expansion rectal siégeant à 5cm de la marge anale, étendu sur 2cm, bien centré, sans distension d’amont mais s'accompagnant d'un retard d’évacuation. Une échographie abdominale, réalisée dans le cadre du bilan malformatif n’a pas révélé d’anomalies rénales, ni de masse pré-sacrée.

## Discussion

Le syndrome de régression caudale est un syndrome malformatif rare associant à des degrés variables une agénésie des vertèbres sacrées et coccygiennes, avec raccourcissement des fémurs, et anomalies gastro-intestinales, génito-urinaires et cardiovasculaires. L’étiopathogénie de cette anomalie n’est pas univoque, cependant la relation avec le diabète maternel est bien établie. Le diagnostic prénatal repose sur l’échographie morphologique par la détermination de la longueur cranio-caudale et de la longueur fémorale. L’imagerie est primordiale pour le diagnostic, mais également pour la décision thérapeutique [2]. L’agénésie sacro-coccygienne y est souvent associée à une agénésie des vertèbres lombaires ainsi qu’à des anomalies du cône médullaire mieux explorées par l’IRM [3]. Des anomalies gastro-intestinales doivent être recherchées telles que l’imperforation anale, l’atrésie ano-rectale, œsophagienne ou duodénale. Les anomalies urologiques à type d’agénésie, dysplasie ou d’ectopie sont pourvoyeuses de complications notamment les infections urinaires à répétition qui peuvent engager parfois le pronostic fonctionnel et vital [4]. Dans notre cas, ce syndrome a été associé à un rétrécissement focal du bas rectum avec signes de dysfonctionnement du mécanisme la vidange rectale. L’échographie abdominale a permis d’éliminer le syndrome de Currarino caractérisé par l’association d’une agénésie sacro-coccygienne, à une malformation ano-rectale et à une masse pré-sacrée [5].

## Conclusion

Le syndrome de régression caudale couvre un ensemble de malformations congénitales orthopédiques, gastro-intestinales, génito-urinaires et neurologiques. Sa relation étroite avec le diabète maternel est bien établie, d’où l’intérêt d’un diagnostic prénatal précoce pour une prise en charge adéquate.

## Conflits d’intérêts

Les auteurs ne déclarent aucun conflit d'intérêts.
